# Innovative podiatry practice: an immersive VR surgery simulation with bimanual haptic interaction

**DOI:** 10.1007/s11517-025-03500-0

**Published:** 2026-02-19

**Authors:** Jason Abounader, Bryan Caldwell, Mark Hardy, Jill Kawalec, Kwangtaek Kim

**Affiliations:** 1 Department of Computer Science, Kent State University, Kent, OH 44242 USA; 2 College of Podiatric Medicine, Mark Hardy, Kent State University, Independence, OH 44131 USA

**Keywords:** Surgical simulation, Haptic interface, Podiatry, Portable system, Virtual reality

## Abstract

**Background/Purpose:**

Researchers and medical experts devised a virtual reality (VR) force feedback system to simulate ingrown toenail removal as a stepping-stone towards a new, immersive form of learning material. The fusion of VR and haptic technologies is an innovative approach to stimulate visual and kinesthetic human senses for learning engagement.

**Method:**

Our bimanual haptic feedback system, tuned with the advice of experts, allows users to physically interact with a 3D deformable virtual foot and perform surgery with various tools, tackling the shortcomings of existing surgical simulations tools in a portable system. The graphic and haptic rendering techniques to simulate each step of the surgical procedure are described.

**Results:**

The usability and effectiveness were tested with 37 participants, including both podiatric medical students and non-medical students. Medical students improved completion time in all surgical tasks by over 160%. Statistical analysis indicates a significant difference in skill of medical students and non-medical students to establish a baseline correlation between performance and experience suggesting preliminary system usability.

**Conclusion:**

Post-simulation assessment techniques provide insight into necessary improvement areas before launching comparative learning impact study in the future. Nonetheless, the results show a promising direction for using our developed system to improve ingrown toenail removal skills.

**Graphical abstract:**

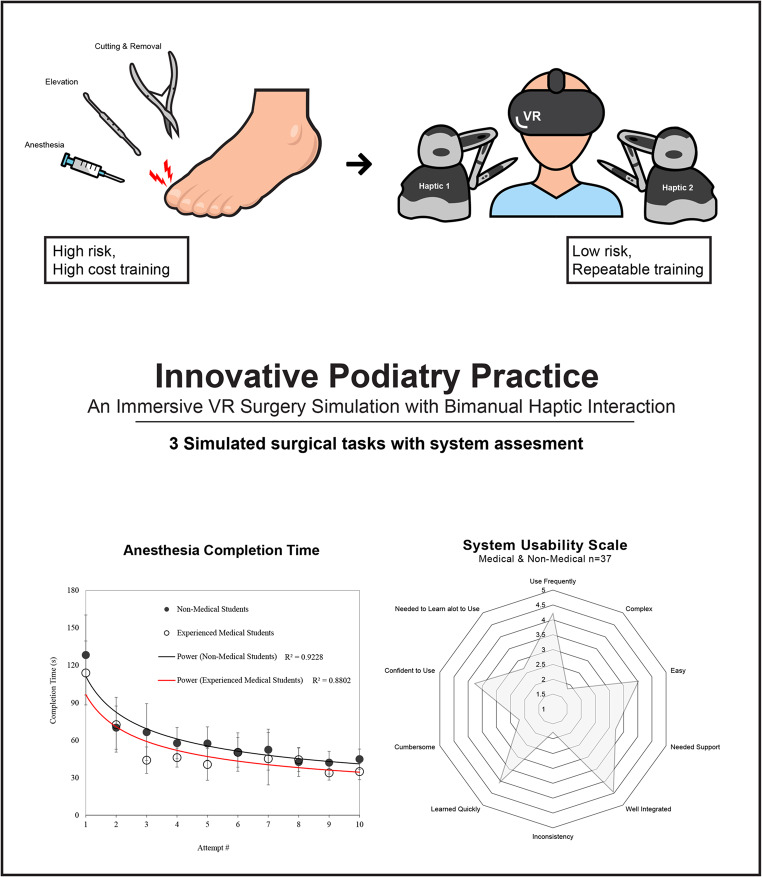

**Supplementary Information:**

The online version contains supplementary material available at 10.1007/s11517-025-03500-0.

## Introduction

One of the first surgical procedures taught to oncoming podiatric medical students, removal of an ingrown toenail, requires proper education of the instrumentation and hands-on learning experience. Also known as paronychia, an ingrown toenail occurs when the corner or side of the toenail grows into the skin causing redness, swelling, and pain to the affected area. The ingrown nail should be removed to relieve the patient of further discomfort. Students typically begin practicing the surgical procedure on a replica toe model made from acrylic, foam, or silicon. Training models like this have been shown to effectively improve perceived confidence before surgical operation [3, 11, 16]. However, while confident, studies have shown that these low-fidelity models do not provide realistic tactile and kinesthetic feedback necessary for mastering psychomotor surgical hand skills [23, 30, 33]. So, eventually students may reinforce their learning with practice on a cadaver, a high-fidelity simulation, for a more realistic affirmation [23]. With great success, podiatric medical residents find the cadaver lab as one of the most strongly beneficial training resources [7]. Yet, the availability of cadavers is limited and requires significant resources to maintain. Cadavers are embalmed with preserving chemicals such as formaldehyde, transported in temperature-controlled units, and housed within a sterile environment leading to many associated fee for medical training institutions [23]. It requires significant resources to maintain, and the cadaver or model is not reusable. Additionally, there is a growing emphasis on safety restrictions, work hours, and cost-saving measures that further reduce traditional practice time for students [13]. Medical institutes are exploring simulations including virtual reality as a possible training tool adjunct or alternative to using cadavers and animals, which could improve training frequency while reducing costs [26, 27]. There has been a movement to simulate surgeries using virtual reality (VR) or haptic feedback in other medical fields [4, 18, 32]. Prior works in surgical simulation have shown that VR systems with head-mounted displays provide higher immersion and presence when compared to non-immersive or desktop display methods [14, 28]. A clear gap exists in terms of practical simulation use in a clinical setting for foot and ankle surgical training, including natural interactions using both hands, and realistic haptic feedback that can replace a cadaver. In this study, we introduce a pilot VR-haptic simulation for ingrown toenail remove practice and explore the preliminary system usability with medical students.

As previously mentioned, tactile experiences in a clinical environment are essential for developing the psychomotor skills required for surgical procedures [30]. These experiences involve the use of both hands to manipulate surgical tools [36], with the applied force varying according to the stiffness of different diseased areas or organs in the foot. The diverse and critical tactile sensations needed to enhance hand motor skills for mastering foot surgical techniques can only be effectively conveyed through efficient and realistic force feedback technology in the development of a VR surgery simulation system. To the best of our knowledge, no studies have addressed this need in podiatry. To fill this gap, we developed a dual haptic interface prototype, integrated into a VR environment on a laptop, to create a realistic clinical setting for simulating an ingrown toenail procedure. We were able to successfully demonstrate pilot usability in our preliminary work [1], which only featured limited system functionality and assessment measured subjectively with a small sample of non-medical participants. We have now expanded our implementation of each surgical task and introduce additional features including user interface, pass/failure feedback, and skin deformation to improve the overall realism and user experience. The extension of this work and its key contributions are summarized as follows:

1. Development and integration of a VR-haptic prototype for a bimanual foot surgery simulation utilizing the Meta Quest Pro VR headset and dual haptic devices (Geomagic Touch) on a portable laptop computer.
2. Realistic haptic (force and tactile) feedback for dynamic interactions with virtual surgical tools and a virtual 3D patient model (foot and toe), developed and verified by medical experts (podiatrists).
3. An interactive surgical simulation scenario for practicing three subtasks (anesthesia, elevation, cutting and removal), which can be extended to other scenarios or applications requiring hand motor skills.
4. A user study with 37 participants, medical and non-medical, to measure the system usability with both qualitative and quantitative assessment.

The remainder of this paper is organized like so: Sect. 2 reviews related studies in surgical simulation. Section 3 details the development of a bimanual VR-haptic interface simulating the ingrown toenail surgical procedure, and then presents our evaluation study, measuring the system’s preliminary usability and effectiveness. In Sect. 4, we present the results and then discuss the implications in Sect. 5, and hereby conclude in Sect. 6.

## Related works

### Podiatric surgery simulation training

We first evaluate physical, real-life practice models as the contemporary standard in foot and ankle clinical training. Jastifer et al. [16] found a positive result when observing a 3D printed foot model as a preoperative planning measure to repair a patient’s long-term bone deformity caused by a motorcycle accident. The study suggests the approach for surgeons even with little computer experience. A clear advantage is shown from pre-surgery simulation training in the field of podiatry. Banwell et al. [3] investigated the perceived effectiveness of foot practice models in education. The experimental group was exposed to the 3D foot model to practice debridement to remove calluses and other markings. The control group was taught through the standard method by debriding a soap bar under guidance of a medical expert. Both novice and experienced groups were able to improve their self-confidence and self-efficacy while decreasing anxiety with the new training method over the standard method. The result of the study enforces the positive effect of simulated, low-risk, clinical skill training. With low-risk training clearly established as a beneficial outcome, we seek to enhance the advantages of simulation training by transitioning to a VR environment, a means more cost-effective long-term than using physical materials. Thus, now we focus on the pre-existing works for VR podiatry training. Labovitz and Hubbard [24] pioneered the movement towards potential virtual supplemental learning material in podiatric medical school. The report describes the effort of Western University College of Podiatric Medicine to develop the first podiatric VR environment. The user is presented text prompts in the virtual space that provide informational descriptions of tools and surgical procedures. Students are assessed to recall the information they learn through testing modules within the system. Another work proposes to implement a virtual environment for foot and lower limb education. Riva et al. [34] conceptualized a VR simulation to train students to diagnose the complications of the diabetic foot amongst other diseases. Users should be able to examine the virtual patient and make an appropriate diagnosis. Although these are early steps towards virtual foot and ankle educational material, both proceeding works are only intended to provide general education without physical haptic surgical interaction, which underscores the existing absence of interactive haptic feedback for podiatry psychomotor skill development.

### Virtual surgery training beyond podiatry

There happens to be an absence in existing immersive virtual podiatric foot and ankle surgical simulations incorporating both the virtual reality headset and haptic feedback at the current moment, but some precedent works exist for other medical fields. In this section, we acknowledge comparative approaches to virtually simulate and evaluate surgical training outside of podiatry but involving similar ingrown toenail procedures, including anesthesia, elevation, cutting, and removal. The Geomagic Touch X 3DOF haptic device was employed in a central venous catheterization simulation [31]. A linear piece-wise force model for needle insertion was applied to simulate the feeling of penetrating the vein. In the user study, medical residents of varying skill levels were able to increase their score, dependent on characteristics of their insertion, over the span of three days demonstrating a learning impact. No VR headset was included. Our approach expands the objective measurement by including time and pathlength as factors when considering novel hand skill improvement. Sadeghnejad et al. [35] quantitatively evaluated their 3DOF haptic endoscopic sinus surgery simulation by comparing the participant performance from training to testing. Their user assessment showed positive improvement in time, force, hand-distance traveled, and success percentage, and therefore, also supports the claim that virtual surgery simulation has a positive impact on surgery skill before partaking in the real thing. The simulation did not include a VR headset either. We intend to improve the immersion with full virtual reality. Another work reinforced the advantage of virtual simulation training. Medellin-Castillo et al. [29] compared the technical skill of sample groups performing orthognathic surgery on a 3D-printed model with pre-surgery practice using haptic-virtual training, virtual training without haptics, and no virtual training at all. The researchers found that participants performed the best after training on the virtual simulation with haptic feedback beforehand versus without. Because users experienced such benefit to their surgical technical skill, the study additionally supports that virtual simulation training is advantageous to increase training repetition whilst decreasing consumed training resources.

With the effectiveness of virtual clinical simulation training established, we can examine technological innovation and notable user evaluation approaches. Di Vece et al. [9] demonstrates pilot applicability of a Veress needle placement simulation. The system trains users to puncture the abdominal wall correctly while experiencing tactile feedback via the 3DOF Geomagic Touch Device. The study focuses on comparing implementation methods: one created with OpenHaptics, the other with Chai3D. Researchers compared insertion error, time, and system usability scale (SUS) results between programs. Users found the OpenHaptics version to provide a better framerate and response than Chai3D, but both systems were rated generally usable from the SUS result. Our podiatric simulation utilizes the OpenHaptics plugin to integrate with Unity3D to provide haptic feedback. These underlying haptic development components have been utilized before. Kim et al. [19, 20] combined virtual reality and haptic feedback into an image-based skin tumor surgery training simulation created in Unity3D with the OpenHaptics plugin. This is the first mentioned related works to include the virtual reality headset. A unique haptic rendering approach was introduced. The feeling of incising into the skin to remove the tumor was created by forming 50 penetrable levels with individual haptic surface properties to represent the epidermis, dermis, and subcutaneous skin tissue layers. A preference for haptics over no haptics was found and the medical group performed better than the non-medical group. Kim et al. [18] found significant differences in objective performance between novices and experts in a mixed reality (MR) haptic needle insertion simulation. We applied a different haptic model to simulate the insertion of the needle, and we use another pen-stylus haptic device to act as the virtual guiding hand rather than a free-floating haptic glove with no translation haptic feedback to support the user while grasping. The study provided shows a clear distinguishment between novice and expert users based on operation time and instrument travel distance. We intend to also demonstrate differences in performance based on user skill to establish the baseline skill-to-performance relationship. Alkadri et al. [2] used path length to assess a minimally invasive spine operation simulation. However, the objective assessment did not consider improvement over time. Kim and Woo et al. [21, 37] assessed the performance of a needle insertion simulation via success criteria. These previous works serve as an example so that we can encompass a wide range of metrics to evaluate user performance and the usability of the surgery simulation between medical and non-medical students.

Additional key strengths can be recognized from the mentioned works such as dynamic haptic rendering, comparative testing procedure, and evaluation metrics. We undertake application of these strengths to the area of virtual podiatry training for an all-inclusive learning system. We combine 3DOF haptic feedback and 6DOF motion tracking paired with a state-of-the-art virtual reality headset (Meta Quest Pro) for the most immersive user experience.

## Method

Removal of the ingrown toenail, wedge excision, begins with application of an anesthetic solution injected into four specific positions around the big toe, a medical procedure known as the digital ring block. Following this numbing procedure, a freer elevator tool is used to release the affected portion from skin and soft tissue to remove restrictions. This is performed at the hyponychium, the distal portion of nail, and at the eponychium, the proximal nail fold. Afterwards, the ingrown portion may be excised with an English Anvil. Any remains, proximate to the nail fold, are severed with a 62 blade. The ingrown portion is removed with a straight hemostat by twisting towards the nail and dropping the hand to release. Within this work, we have successfully deployed techniques to simulate the digital block anesthesia, elevation of soft tissue, cutting with the English Anvil, and novel removal of the ingrown portion to finish.

**Fig. 1:**
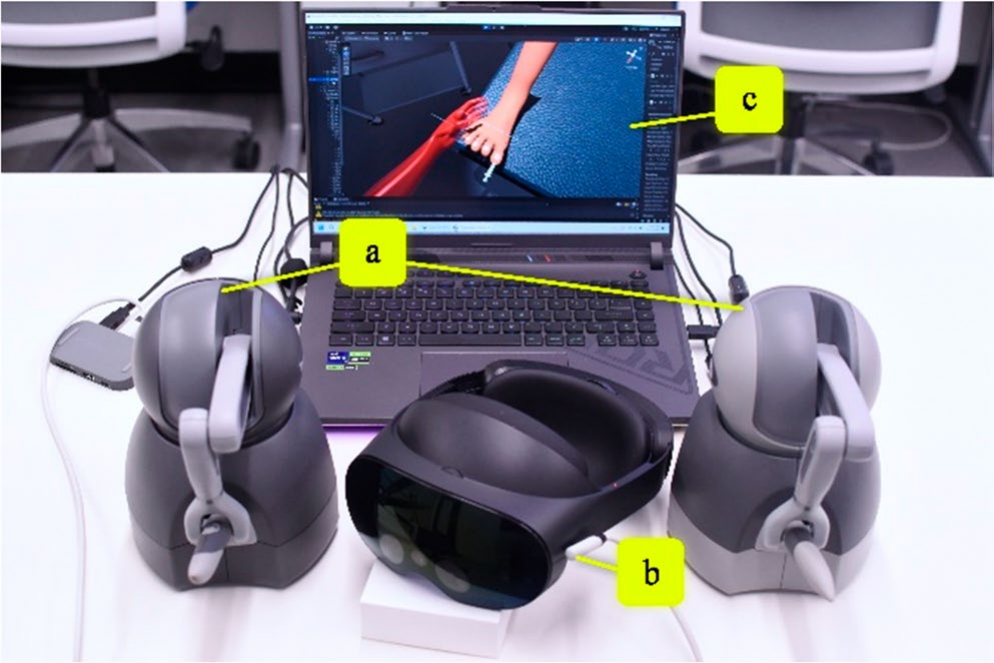
An image of the system setup. (**a**) two haptic devices placed approximately shoulder-width. (**b**) Meta Quest Pro VR display headset for immersive view. (**c**) High-performance computing laptop to execute the simulation application. All devices connect to and communicate with the laptop, hosting the surgery simulation, via USB connection. The laptop screen provides a preview of the environment explored within the VR headset

The following section presents the development to simulate the described podiatric surgical tasks in virtual reality. We describe each individual component in their respective subsections which ultimately conjoin to provide an immersive, surgical training experience: the virtual reality (VR) interactive graphical rendering framework, haptic feedback rendering utilizing dual haptic devices, and then the implementation of an ingrown toenail removal procedure from the combination of the two components. The system is portrayed in Fig. 1.

### VR interactive graphical rendering framework

Built upon Unity3D Game Engine Version 2023.3.14f1, we integrated virtual reality through the Meta XR All-in-One SDK. The Meta Quest Pro (1800 × 1920 per eye, 90 Hz, 60 mm FOV) VR headset is worn to experience the simulation. The immersive environment, viewed from inside the VR headset, places the user inside a virtual operating room. The virtual scene contains an operating room, table, human foot, virtual hand, and surgical tools. The foot and surgical tools were 3D modeled from reference images in Blender. The room was obtained from the Unity3D Asset Store. See Table 1 for the distribution of mesh vertices. Geometry is rendered through the Universal Render Pipeline (URP) configured with effects to improve user visual appeal.

**Table 1:** Virtual simulation mesh geometry

Environment Vertices					
Foot	Hand	Operating Room			
15,009	5,045	62,997			
Surgical Tools Vertices					
Syringe	Freer Elevator	English Anvil		Straight Hemostat	62 Blade
986	13,931	2,756		12,592	27,254

Total 140,570 vertices

**Fig. 2:**
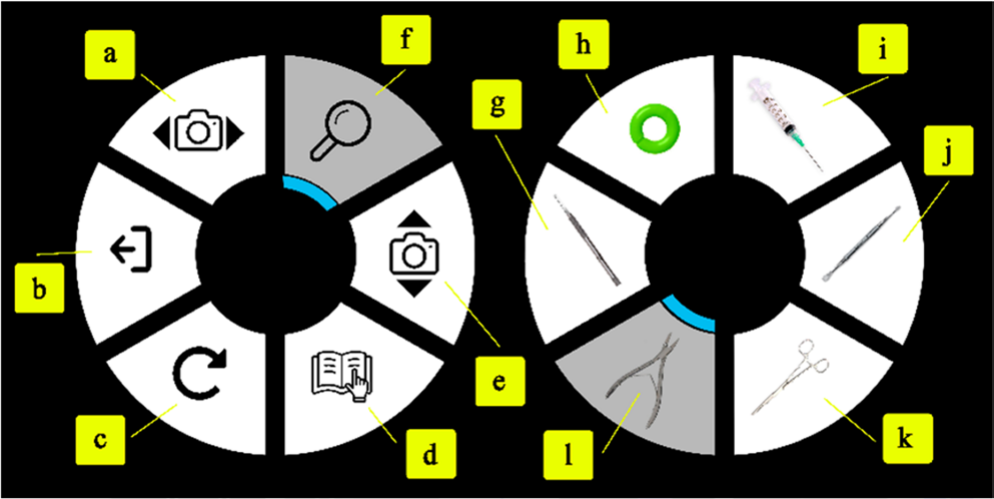
The left- and right-hand user interface viewable in VR. The user presses a stylus button to activate each interface and twists the stylus pen to rotate the blue cursor, cycle through the options, and select the desired choice. The functions of the interface: (**a**) VR camera swivel, (**b**) exit simulation, (**c**) reset toenail, (**d**) hint, (**e**) VR eye-height adjust, (**f**) VR zoom, (g) 62 blade tool (unused), (**h**) apply tourniquet, (**i**) syringe, (**j**) freer elevator tool, (**k**) straight hemostat, and (**l**) English anvil

When introducing new technology, it is imperative to include intuitive user interface (UI) design. In this case, users must switch between surgical tools and adjust their VR view angle of the patient’s foot, on demand, while operating with the dual Touch haptic devices. Navigating between viewing angles and tool selection is made simple with the design of two dial-based graphical user interfaces, one for each hand, summoned by pressing a button on the haptic stylus. Refer to Fig. 2 for details on the appearance and functionality of the interface. Text dialogue and tactile feedback (to be discussed) provides guidance for the novice user, these attention cues are proven to improve task performance in VR [17]. The dial interface is an improvement from our initial concept shown in previous work [1], where surgical tools and view angles were accessed by cycling through each option, one button press at a time, until the desired option appears. A pilot user study was conducted to measure the usability between the new dial interface and the previous iterative design. 5 participants were placed inside the simulation and asked to select each surgical tool and camera angle in randomized order per participant for each UI design. Though users took 1.8x longer to select the correct option with the dial interface, in a NASA TLX assessment [12], users rated lower perceived mental, physical and temporal demands as well as lower frustration and effort using the dial interface (µ = 6.13) than the iterative method (µ = 7.53). Additionally, user comments favored the enhanced feeling of control from the dial interface. Thus, we are inclined to choose the dial interface as the improved tool-selection option with user experience and lower perceived workload as priority over selection speed.

We describe a novel approach to simulate skin deformation when pressure is applied to the virtual foot to enhance perceived realism both visually and kinesthetically. Algorithm 1 outlines our mesh deformation method. Let each vertex node **V**_**i**_ = [x_i_, y_i_, z_i_]_local_ ∈ ℝ^3^ contain a 3D point for all vertices of the foot mesh. Let a triangle node **T**_**i**_ = [**V**_**i**_, **V**_**i+1**_, **V**_**i+2**_]_local_ ∈ ℝ^3^ contain three vertex nodes that make up each mesh triangle. Upon contact with a mesh triangle, mass-spring-damper physics are applied to each vertex node associated with the triangle. The force is dispersed across neighboring vertex nodes under the inverse square law $\:\overrightarrow{\mathbf{F}}=1/{d}^{2}$ where *d* is the distance from the original point of contact with the haptic device. To avoid dispersing force endlessly through all connected nodes of the mesh, a flag is defined and a minimum force threshold must be met for a node to transfer force to another. Displaced nodes are added to an update queue where spring force (6) is applied every timestep to return vertex nodes to the original position. Once a displaced vertex node is marginally close to its original position it is removed from the update queue. Deformation of vertices is processed at 1Khz on the physics thread for real-time response with haptic collision, yet the visual mesh is updated on the graphics thread since a lower frequency is minimally required for adequate human visual perception [8, 22]. These optimizations are necessary for real-time, responsive deformation. Collision detection will be discussed in the following haptic rendering subsection.

**Algorithm 1** Skin Mesh Deformation


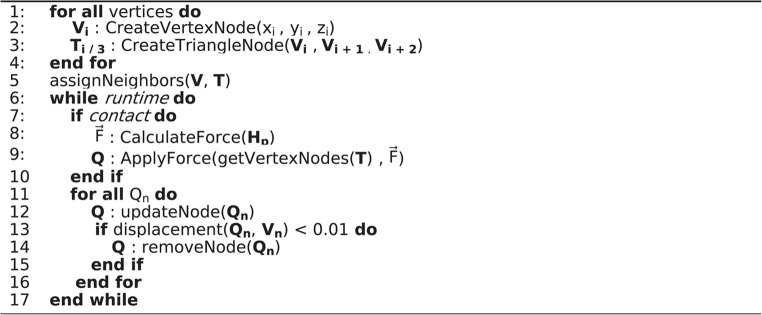


**Fig. 3:**
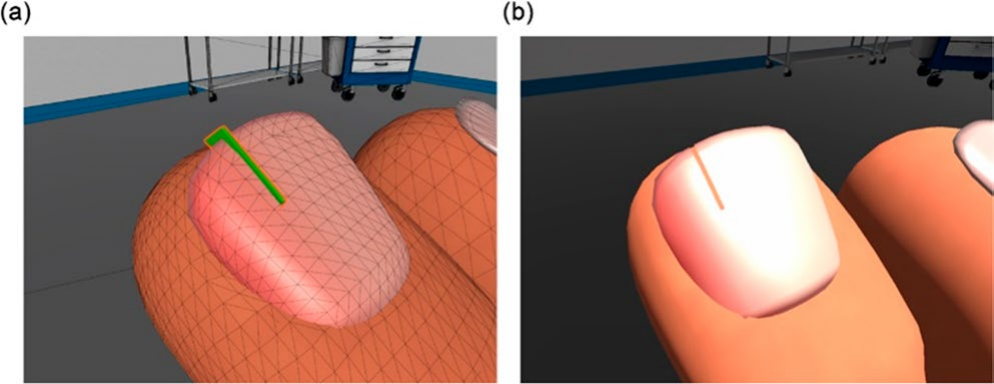
A shader masking approach to simulate cutting of the ingrown nail. (**a**) A geometric object is spawned upon user cut action represented in green. (**b**) The final effect visible to the user. The invisible geometry masks the toenail creating the illusion of cutting

A present challenge in virtual surgical simulation is effectively rendering mesh cutting at a real-time interactive rate. Boothroyd et al. [5] investigates several methods particularly for virtual podiatric surgery purpose including Boolean Subtraction and planar slicing. We avoid the complexity and computational demand of direct mesh modification by shifting towards a shader masking approach. When the user initiates a cutting action, a geometric mesh object is instantiated at the surgical tool position and orientation with unique material properties as shown in Fig. 3. The render order is modified to always render the mask before the toenail object. The surface shader is assigned property “Pass {Blend Zero One}” which denotes a transparent object while rendering geometry behind it. The space of the cut area appears completely void from this shader field. The overlap of the transparent cutting area object with the toenail mesh effectively represents the visuals of a cut for this particular use case. The masking effect is reutilized to illude removal of ingrown toenail portion the user has cut.

### Integration with bimanual haptic rendering

To wield and operate with the virtual surgical tools, the user is equipped with two stylus-based Geomagic Touch haptic devices (3D Systems Inc.; 3.3 N Max; 3DOF; 431 × 348 × 165 mm), one for each hand. Each stylus pen contains two buttons required to control the VR simulation. Our system asynchronously communicates with the Touch devices through the OpenHaptics Plugin [15] on the physics thread at a fixed rate of 1Khz, separate from the graphics thread, to send instantaneous instruction necessary for real-time haptic feedback [8]. With medical expert-guided feedback, we adjusted haptic surface contact properties parameters as shown in Table 2.

**Table 2:** Tool-to-Skin haptic contact properties (Unitless effect magnitude [0, 1] in OpenHaptics)

	Stiffness	Static Friction	Dynamic Friction	Damping	Pop-Through
Syringe	0.2	0.5	0.35	0.1	0.034
Freer Elevator	0.2	0	0	0.1	0
English Anvil	0.2	0.3	0.3	0.1	0
Straight Hemostat	0.2	0.25	0.25	0.1	0

**Fig. 4:**
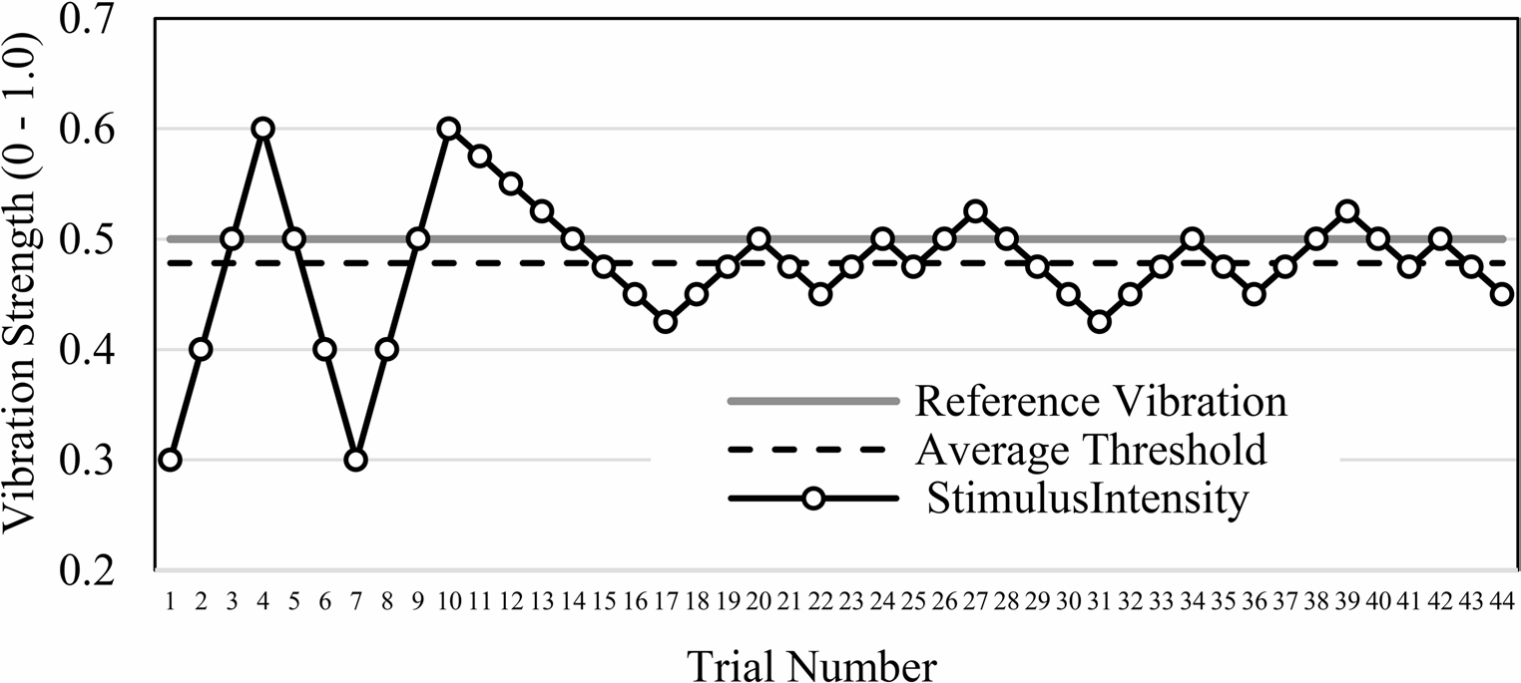
Sample adaptive staircase for minimally perceived vibration strength for tactile feedback

We take advantage of the capability of the haptic devices to provide vibrational tactile feedback to enhance user experience within the simulation. Uses will experience 200 Hz vibration for 100ms as an affirming response while navigating the dial selection interfaces. The strength of the vibrational feedback (0.25) was devised from conducting a pilot study to find the minimum recognizable vibration magnitude from user’s perception via the adaptive staircase method [25]. 3 participants were situated in a quiet room, wielded the stylus of a Geomagic Touch Device, and asked to signify which vibration felt stronger from two 10ms samples in random order. One vibration of 0.5 magnitude served as reference and was constant during the study. The other began at 0.3 and adaptively climbed or descended each trial based on each answer. The adaptive test was configured to have three reversals with 0.1 increments and then small increments of 0.025 for twelve reversals. See Fig. 4. for a sample of a participant’s vibration adaptive staircase result. The minimally perceived vibration strength was 0.045. Thus, any vibration should differ by this scale to be humanly perceptible. The vibration tactile feedback may be utilized to affirm other user actions in a future iteration.

To simulate the feeling of puncturing into the skin with the anesthesia needle, we apply a linear force model where the needle path is represented as a 3D line (1) from the initial point of penetration $\:\overrightarrow{{p}_{0}}$ in the insertion direction $\:\overrightarrow{d}$. The net force $\:\overrightarrow{{F}_{NET}}$ (3) experienced is the sum of penetrating force $\:\overrightarrow{{F}_{pen}}$, the resisting force from pressing in and pulling out from the skin, and path force $\:\overrightarrow{{F}_{path}}$, the force guiding the needle to remain on its trajected path. The needle depth inside the skin (2) is represented as a vector $\:\overrightarrow{D}$ which is the result from the subtraction of the initial $\:\overrightarrow{{p}_{0}}$ from the current position $\:\overrightarrow{{p}_{c}}$. The penetrating force (4) is implemented as a piecewise function. A viscosity force is applied for needle insertion derived from Gerovich et al. [10] who applied the function in one dimension. This force is the product of the damping coefficient $\:b$ (0.2), the needle depth, and the velocity in the y-direction. A stiffness equation is applied to restrain the needle from penetrating any further than 30 mm. Stiffness coefficient $\:{k}_{1}$ is set to 0.6. The path force (5) is derived from finding the orthogonal vector from the current position $\:\overrightarrow{{p}_{c}}$ back to projected insertion path $\:\overrightarrow{p}\left(t\right)$. The vector is used to apply a spring force with coefficient $\:{k}_{2}$ (7.9) to maintain the needle on path. Damping is applied with coefficient $\:c$ (0.15) on the XZ plane orthogonal to the insertion direction to dismiss oscillating forces. The values of the coefficients were agreed from the opinion of podiatric medical expertise. Next time we intend to align the values using force overtime recordings of the real surgical operation.

1 $$\:\overrightarrow{p}\left(t\right)=\overrightarrow{{p}_{0}}+\overrightarrow{d}t$$

2 $$\:\overrightarrow{D}=\overrightarrow{{p}_{c}}-\overrightarrow{{p}_{0}}$$

3 $$\:\overrightarrow{{F}_{NET}}=\overrightarrow{{F}_{pen}}\left(\overrightarrow{D}\right)+\:\overrightarrow{{F}_{path}}$$

4 $$\:\overrightarrow{F_{pen}}\left(\overrightarrow D\right)=\left\{\begin{array}{c}b\times\left\|\overrightarrow D\right\|\:\times\:\overrightarrow{v_y},\:\:\left\|\overrightarrow D\left(t\right)\right\|<30\\\:-k_1\ast\left(\left\|\overrightarrow D\right\|-30\right),\:\left\|\overrightarrow D\left(t\right)\right\|\:\geq\:30\end{array}\right.$$

5 $$\:\overrightarrow{F_{path}}\left(t\right)=k_2\times\:(\parallel\overrightarrow d\times\:\overrightarrow D\parallel/\parallel\overrightarrow d\parallel)\times\:\left(\overrightarrow D-{proj}_{ \overrightarrow d}\overrightarrow D\right)-\:c\times\:(v_x,0,v_z)$$

We apply the Hooke’s Law Spring model to simulate the piercing of soft tissue, grasping with the left virtual hand, and restraining the English Anvil tool whilst cutting. The property of vector subtraction was utilized to create a spring force vector attempting to return the current position $\:\overrightarrow{P}$ to the original point of contact $\:\overrightarrow{{P}_{0}}$ as shown in (6).

6 $$\:\overrightarrow{{F}_{spring}}=-k*(\overrightarrow{{P}_{0}}-\overrightarrow{P})$$

**Fig. 5:**
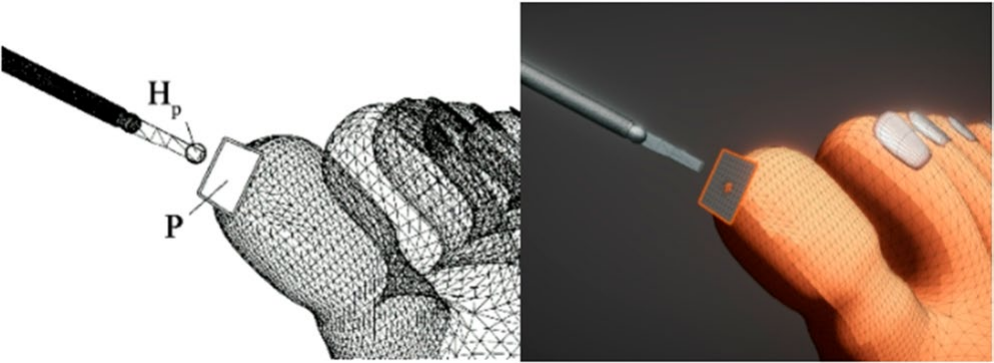
The interpolating plane **P** serves as the collider for the haptic point **H**_**p**_ positioned at the end of the surgical tool. The collider is not visible during runtime, but users will experience the contact

An abstract system has been developed to account for common performance and accuracy issues when calculating collisions with a dynamically deformable, non-convex mesh for force feedback. We propose a plane collider mapping approach where, for every physics timestep, the plane collider is mapped to the nearest mesh triangle to the position of the user’s surgical tool and serves as the colliding object for that interaction. Figure 5 visualizes the tool-to-collider interaction.

The plane mapping position is determined by solving the closest node to a point in a 3D space problem. Storage of the mesh triangles **T**_**i**_ under the octree data structure provides fast access in $\:O(\mathrm{log}N)$ time complexity where *N* is the number of triangle nodes. It is assumed that triangle nodes remain within their octant when deformed to avoid rebuilding the octree during runtime We search the defined octree for the nearest node by octant in $\:O(k+\mathrm{log}N)$ where $\:\mathrm{log}N$ is the complexity to visit the correct octant and k is the amount of nodes in that octant. The plane collider is then assigned the position and normal orientation of the determined closest triangle node **T**_**i**_. The movement of the plane between assignments is interpolated over 25ms to remove potential oscillation.

**Fig. 6:**
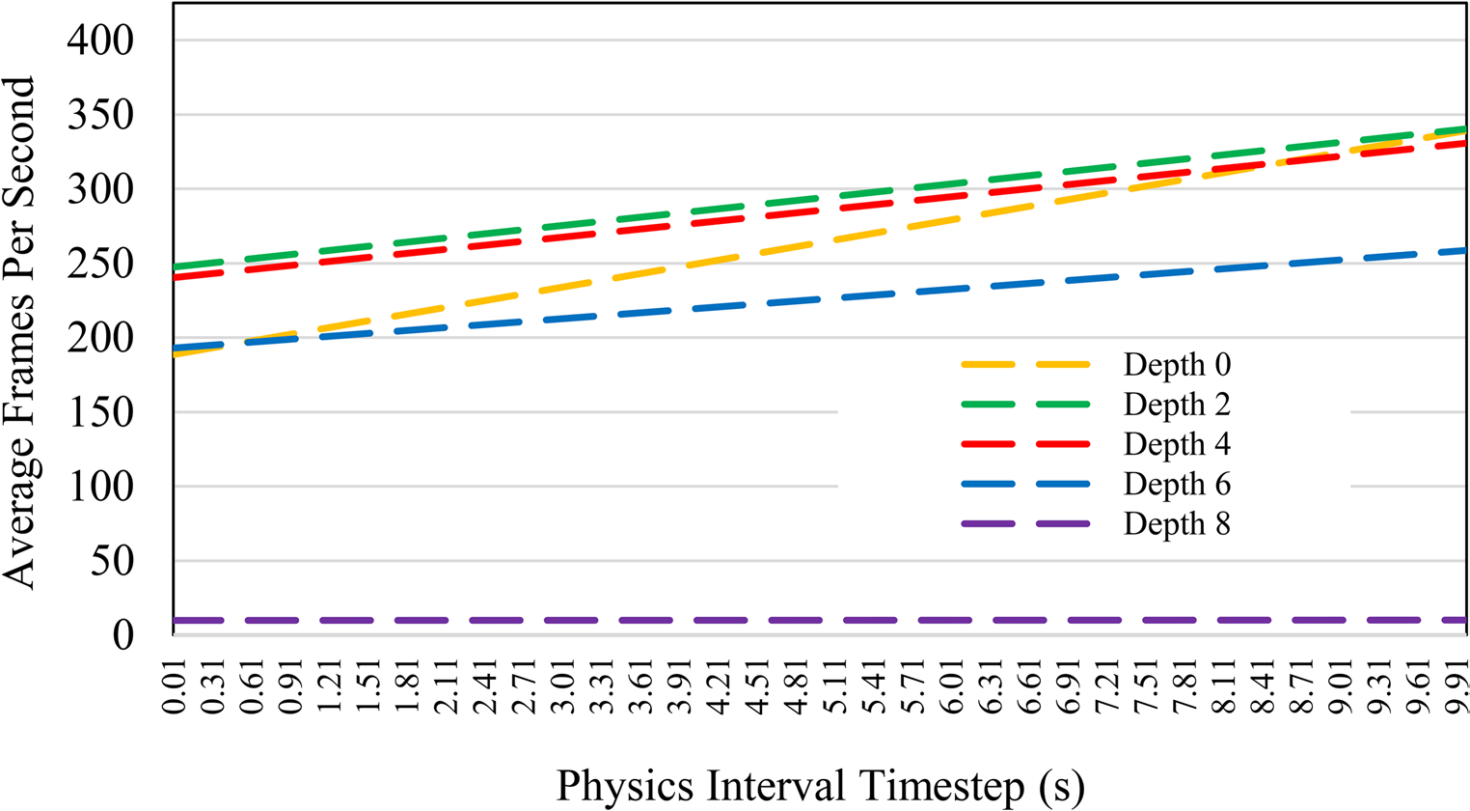
Comparing octree data structures of varying depth levels to find the optimal depth for mesh collision and deformation by measuring performance over the duration of a simulated interaction

An octree of depth level 2 was deemed the best performing fit for the real-time collision with the deformable foot mesh. The performance of octree depth levels was compared by creating an isolated simulation between the virtual foot and a sphere with rigid-body physics properties. The sphere object was dropped onto the foot mesh (4356 vertices) from a defined constant position. The position of the plane collider mapped to the closet mesh triangle and provided collisions until the sphere object rolled off and fell into the void. This procedure creates a consistent and repeatable collision benchmark, to measure octree query performance for each depth level. The simulation was performed three times for each octree depth level 0 to 8. The FPS was recorded and averaged for each physics timestep as shown in Fig. 6.

### Implementation of ingrown toenail removal

**Fig. 7:**
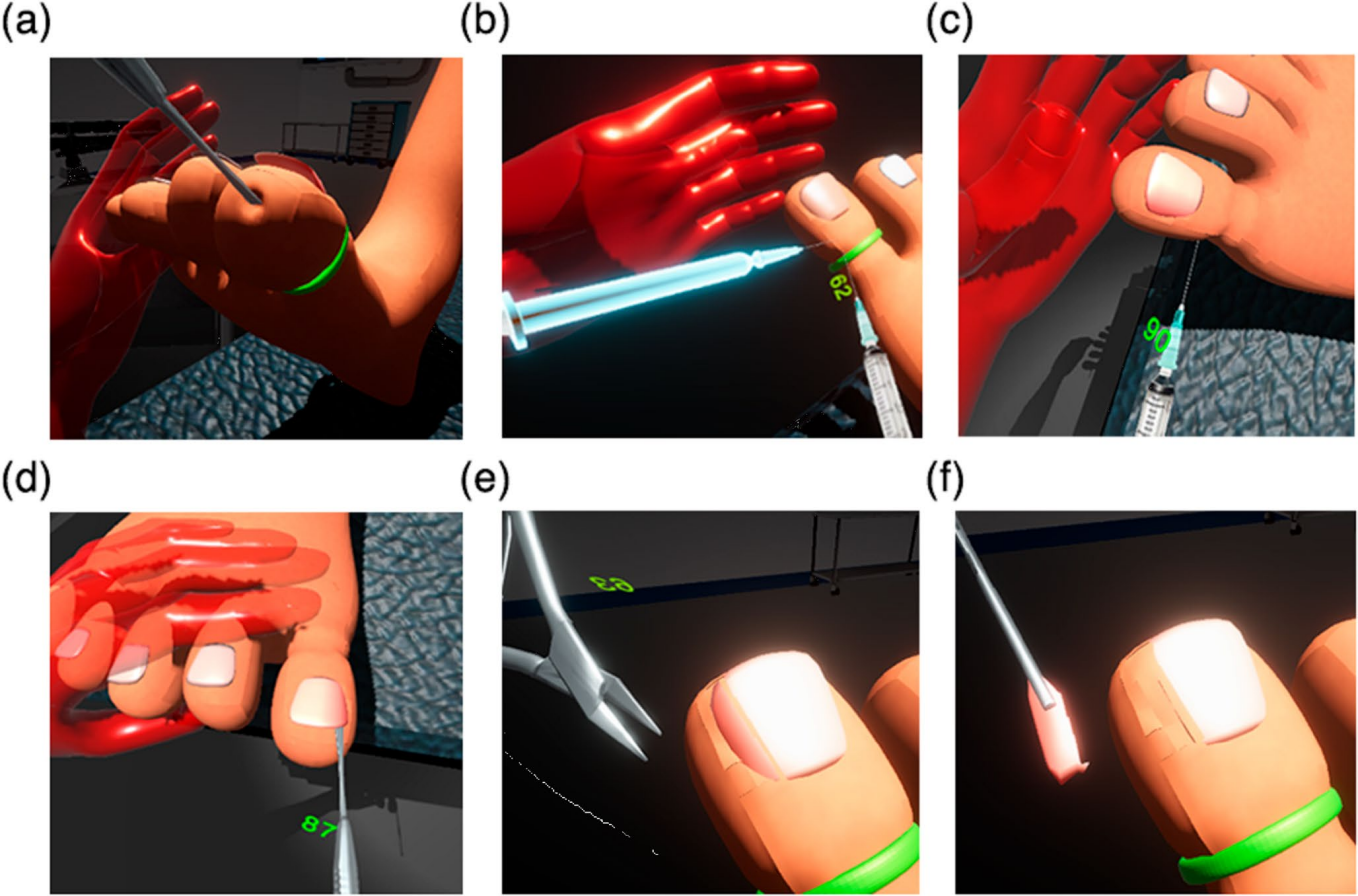
Developments of the simulation system are showcased. (**a**) A demonstration of skin deformation when pressure is applied. (**b**) A virtual hologram tool appears in VR to guide the user for novice learning. (**c**) Virtual application of anesthesia across medial to lateral position. (**d**) Elevation of soft tissue from distal to proximal underneath toenail. (**e**) A successful cut of the ingrown affected portion with the English Anvil. (**f**) Removal of ingrown portion intact with the straight hemostat

The application of anesthesia is performed by injecting into four separate spots with the syringe tool. The needle is constrained to move on the trajected path with the described force model. Figure 7c demonstrates application of anesthesia to the toenail in the virtual environment. We defined success criteria for each surgical subtask. For successful completion of anesthesia, the user must inject into the toe four times at a depth of at least 80% the length of the needle. The insertion angle must be between 70 and 90 degrees orthogonal to the skin. Failing to inject at the correct angle will result in a failure mark for that attempt.

During the elevation task, the surgeon pierces between the skin and nail at the hyponychium and then at the eponychium (Fig. 7d). There is a feeling of pressure and then a quick release as the attachment between the top of the nail and the skin is released. It is important that podiatric students feel the correct amount of pressure to exert while learning this procedure. We have simulated this feeling by presenting the two piercing locations as bounding volumes. The stiffness coefficient $\:k$, set to a value of 2, controls the amount of pressure experienced. As the magnitude of $\:\overrightarrow{{F}_{spring}}$ exceeds our defined threshold 1.815 N, the effect diminishes, and the volume is considered pierced. We defined a successful completion of elevation as a puncture of soft tissue at the defined position of the hyponychium and at the eponychium at an angle between 70 and 90 degrees planar with the nail. This will result in a success mark for that elevation trial. Attempting to puncture outside the angle range results in a failure.

When the operator aligns the virtual English Anvil tool on the toenail and presses the front button on the stylus, a cutting animation is played, and a masking volume is created. The result forms an illusion of cutting the toenail as seen in Fig. 7e. For completion of the cutting and removal subtask, user must perform at least 3 cuts that are at least 70 degrees planar with the toenail. The task ends successfully when the user removes the nail with the grasping forceps while following the criteria (Fig. 7f). Ineffectively severing the ingrown nail portion or cutting at an incorrect angle will lead to a failure mark for this task.

The left-hand stylus appears as a hand within the virtual environment and allows the user to perform grasping. The ability to grasp may provide stabilization and increase coordination while operating [36]. The virtual hand model shifts from the color red to green when the user holds their hand steady. Grasping is activated by holding the front button on the stylus pen. The hand is bound in place via Hooke’s Law (6) with stiffness coefficient $\:k$ is set to 1.5. The grasping status is terminated once the front button is released.

Additional features have been implemented to assist in potential usability for learning. The system is set up with two modes: training mode allows retries and testing mode does not. To aid in training first-time users in the virtual environment, a hint feature was introduced. Upon selection from the UI, a silhouette of the tool appears and follows a pre-recorded attempt that demonstrates proper use of the instrument. This concept has potential to display the hand-tool motion of a medical expert in VR that can be replayed an unlimited number of times. See Fig. 7-b for the appearance of the ghost tool. The system offers immediate visual feedback when a surgical task (anesthesia, elevation, or cutting & removal) is performed incorrectly according to the defined success criteria. There is potential to display additional information such as hand velocity, trajectory, and time.

## Results

In this section, we introduce a user study to assess the usability of the system for virtual surgery training, followed by the subjective results, and then objective results showcasing the performance of participants and potential training applicability. We aim to demonstrate a correlation between skill and user performance by comparing the result of the non-medical students’ group, NM, to the experienced medical students, MED. The mean µ and standard deviation is shown for each objective measure. We apply a heteroscedastic two-sample t-test of unequal variance with 5% confidence interval α and examine the one-tail p-value to find statistical significance between groups.

### Experiment design

A user study was conducted with medical students from the Kent State University College of Podiatric Medicine and additional non-medical students to assess the usability of the VR-haptic system. Participants were requested to perform the virtual anesthesia, elevation, cutting/removal surgical tasks to the best of their ability. we sought to assess user’s subjective perceived workload and usability of the VR simulation using the NASA Task Load Index (TLX) [12] and System Usability Scale (SUS) [6] as post-simulation assessment. Besides subjective information, the following real-time data was recorded: virtual position of each haptic stylus, virtual angle of each stylus, virtual angle of tool in relation to task, force feedback, button presses, tool selected, and trial success or failure.

#### Apparatus

The testing procedure was administered on an Asus ROG Strix G16 Laptop equipped with 13th generation Intel i9-13980HX 4.8hz, Nvidia RTX 4070 8GB GDDR6 VRAM, 32GB DDR5 RAM, and Windows 11 installed operating system. The participants wield the dual Geomagic Touch Devices and wear the Meta Quest Pro VR Headset.

#### Participants

The population sample includes both medical (MED) and non-medical students (NM). The sample is split into two groups. One group is made up of experienced podiatric students (MED), years 2–4, with hands-on experience with ingrown toenail removal surgery taught within the university. Another group consists of 1 st year podiatric students (NM) that have not yet learned the surgical procedure along with non-medical students to grow the power of the study. There is a total of 37 participants between 19 and 32 years of age. The majority has previous experience with virtual reality technology from games or other VR educational opportunities. Few had experience with haptic technology. See Table 3 for a complete breakdown of our two sample groups.

**Table 3:** User evaluation sample groups

	Non-Medical Students NM	Experienced Medical Students MED
Total	23	14
Male	17	5
Female	6	9
Toe Surgery Experience	No	Yes
Prior VR Experience	65.2%	70.0%
Prior Haptics Experience	13%	10%

#### Procedure

**Fig. 8:**
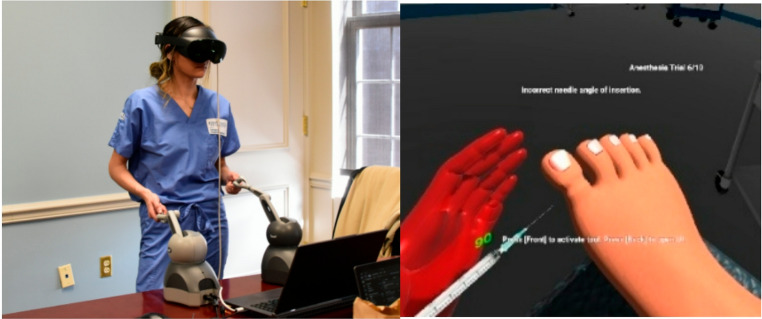
A medical student wears the Meta Quest Pro VR headset and begins the experiment (left). The testing screen shown in VR notifies of a success or failed attempt after each trial (right)

Over a one-hour experiment timeslot, the participant is debriefed on the project details including VR and haptic equipment, research goal, data collected, and risks such as the chance of VR dizziness. They were asked to sign a consent form in correspondence with the Institutional Review Board (IRB) protocol. After signing, the test practitioner wears the Meta Quest Pro VR headset and provides a tutorial to the participant showing the functions within the system and how to do the surgical operation. The participant is given 3 min per tool to practice each surgical task. The participant may reset the toe during this period to retry. After time, the participant is moved to testing mode where they are asked to conduct anesthesia, elevation, cutting and removal for 10 iterations consequently for each task (30 attempts total). We consider cutting and removal together in one task. For each iteration, the user begins by raising both haptic devices above a defined virtual height. The height is visualized in the simulation by a blue level that must be exceeded. If both hands are raised over the level, it turns green to indicate that the trial will begin shortly. The user is to now complete the indicated surgical task. The user is indicated of a success or failed attempt based on the specific criteria previously mentioned. The blue level returns signaling to prepare for the next attempt and the trial is repeated. Upon completion, participants are removed from the simulation and asked to complete the post-simulation assessment. Participants were compensated $20 for their participation (Fig. 8).

### System usability assessment

**Fig. 9:**
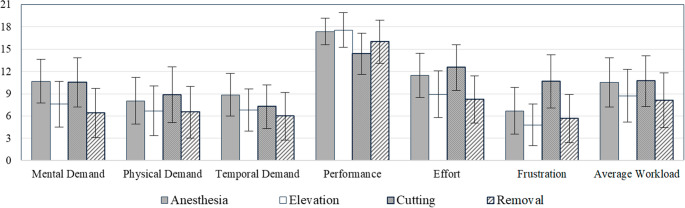
NASA Task Load Index for each virtual surgery subtask of all groups combined. 0 indicates low perception and 21 is high

**Fig. 10:**
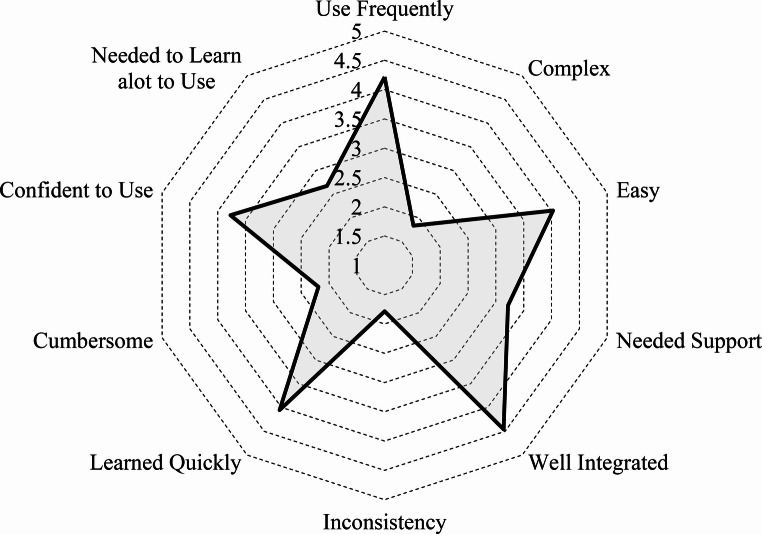
System Usability Scale derived from the post-simulation user assessment of the system. Levels of agreement from low to high (1–5)

We evaluate subjective system usability by observing the ratings of participants through the NASA Task Load Index and System Usability Scale. Given the comparable outcomes observed between the two groups, they were treated as a single cohort in the subjective analysis for additional clarity. In the NASA TLX, the combined result of both non-medical and medical students is illustrated in Fig. 9 for each subtask (µ = 9.52 ± 7.07). Note that the cutting action conditions the context in which removal occurs; one enables the other. These tasks cannot be systematically separated for objective analysis. Nonetheless, we are able to subjectively assess each task in isolation to gather more insightful feedback on the perceived workload. Users report high performance and medium to low perceived demand for all tasks. The radar graph, shown in Fig. 10, depicts the combined SUS result (µ = 3.22 ± 1.35) of both unmedical and medical students. Ranking a mean over 4 out of 5, participants agree in favor of using the simulation frequently, found ease-of-use, and experienced well system integration. Ranking a mean below 2 out of 5, users disagree that the system is complex or inconsistent.

We expanded the user poll by inquiring users for additional information on their experience. Participants were asked to provide a realism rating along with if they would like to continue this type of training in the future. Users rated perceived realism on average 7.2 out of 10. When asked to indicate if they would continue virtual surgery training in the future, 35 of 37 participants indicated “yes”. Participants were allowed to leave a comment at the end of the post-simulation questionnaire. One remark of a medical student, “As someone with very little experience with VR, I felt comfortable using this system only after being shown how it operates. After some practice time, I felt that the system was easy to use, and I think the potential to use it is extremely promising especially for surgical procedures.” Another medical student stated, “With some improvements, I could see this at podiatry schools!” A non-medical student wrote, “The device is really great, as a person not much familiar with VR, I found it difficult to use during initial phase, but later I got used to the technology and I really liked it.” An additional non-medical student closed, “It was a good experience and I’m glad I was able to be part of this research”.

### Objective user performance data

**Fig. 11:**
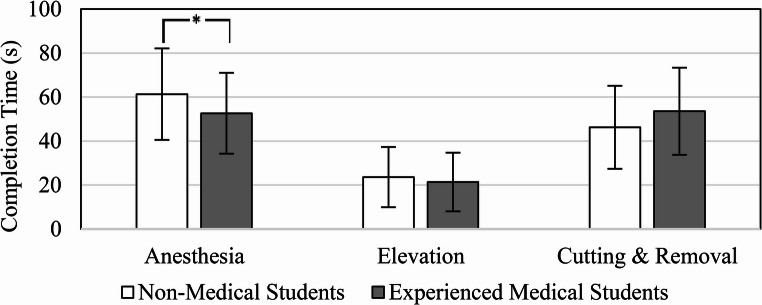
Average time to complete each virtual surgical subtask compared between those who have been educated and practiced ingrown toenail surgery and those who have not. * Signifies a rejection of the equality of means between groups (*p* < 0.05)

We explore user performance as a metric of system usability. It is necessary that medical students perform objectively better than unexperienced individuals within the virtual surgery simulation. Each attempt to complete the virtual digital block anesthesia, elevation of soft tissue, and cutting followed by removal of the affected region was timed and evaluated as a metric of user performance within the simulation. Figure 11 illustrates a discernible difference in the time to complete each surgical subtask between the average non-medical student and the experienced medical student. Experienced medical students took significantly less time to complete the virtual anesthesia than unmedical students (MED µ = 52.60 ± 18.37 s; NM µ = 61.67 ± 20.77 s; *p* = 0.018). On average, medical students completed the simulated elevation subtask sooner than inexperienced non-medical students (MED µ = 21.37 ± 13.29 s; NM µ = 23.61 ± 13.65 s). Additionally, no significance is observed for the time to complete cutting and removal between groups (MED µ = 53.55 ± 19.82 s; NM µ = 46.28 ± 18.87 s).

**Fig. 12:**
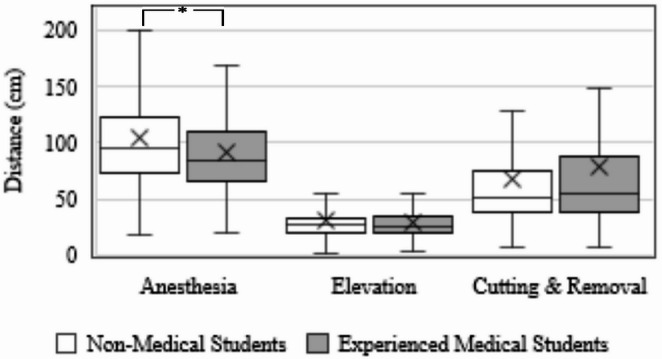
The pathlength of the virtual surgical tool to complete each surgical subtask in the simulation between non-medical and medical groups. * Signifies a rejection of the equality of means between groups (*p* < 0.05)

We sought to investigate the path length, the distance traveled by the participant’s virtual tool to complete each simulated ingrown toenail removal subtask, to see if there was a noticeable gap of spatial efficiency amongst non-medical and experienced medical students. A gap in path lengths can be noticed in Fig. 12. An observation was found that experienced medical students completed virtual anesthesia with a significantly shorter hand-pathlength than non-medical students (MED µ = 92.04 ± 22.34 cm; NM µ = 103.96 ± 23.36 cm; *p* = 0.008). Medical students, on average, were also more route-efficient to complete the virtual elevation (MED µ = 29.48 ± 8.55 cm; NM µ = 29.86 ± 10.28 cm). Yet, medical students exhibited longer hand motion for the virtual cutting & removal subtask (MED µ = 78.11 ± 36.45 cm; NM µ = 67.77 ± 28.10 cm). No statistical significance was found between groups from the latter two surgical subtasks.

**Fig. 13:**
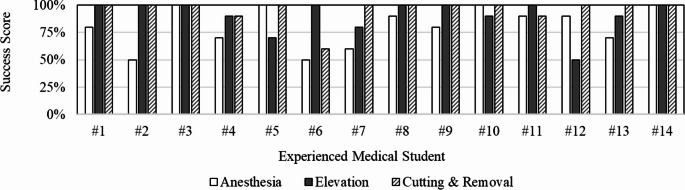
The average success score of individual experienced medical students accumulated from ten attempts for each surgical subtask

From each attempt, a pass or fail mark was assigned based on if the participant was able to correctly follow the defined success criteria establishing a feasible virtual surgical skill scoring system. We present the individual scores of the six experienced medical students performing all three surgical subtasks in Fig. 13. It can be observed how scores may vary amongst experienced students exposing areas that may require more virtual training. We anticipate that more intricate scoring metrics may be defined from the initial pilot design.

### Potential training impact

**Fig. 14:**
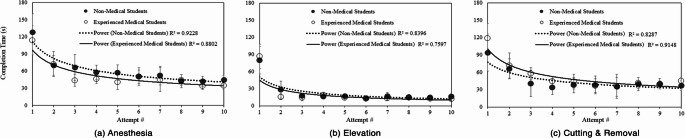
The average time to complete each virtual surgery subtask by consecutive attempt between non-medical and experienced medical students

**Fig. 15:**
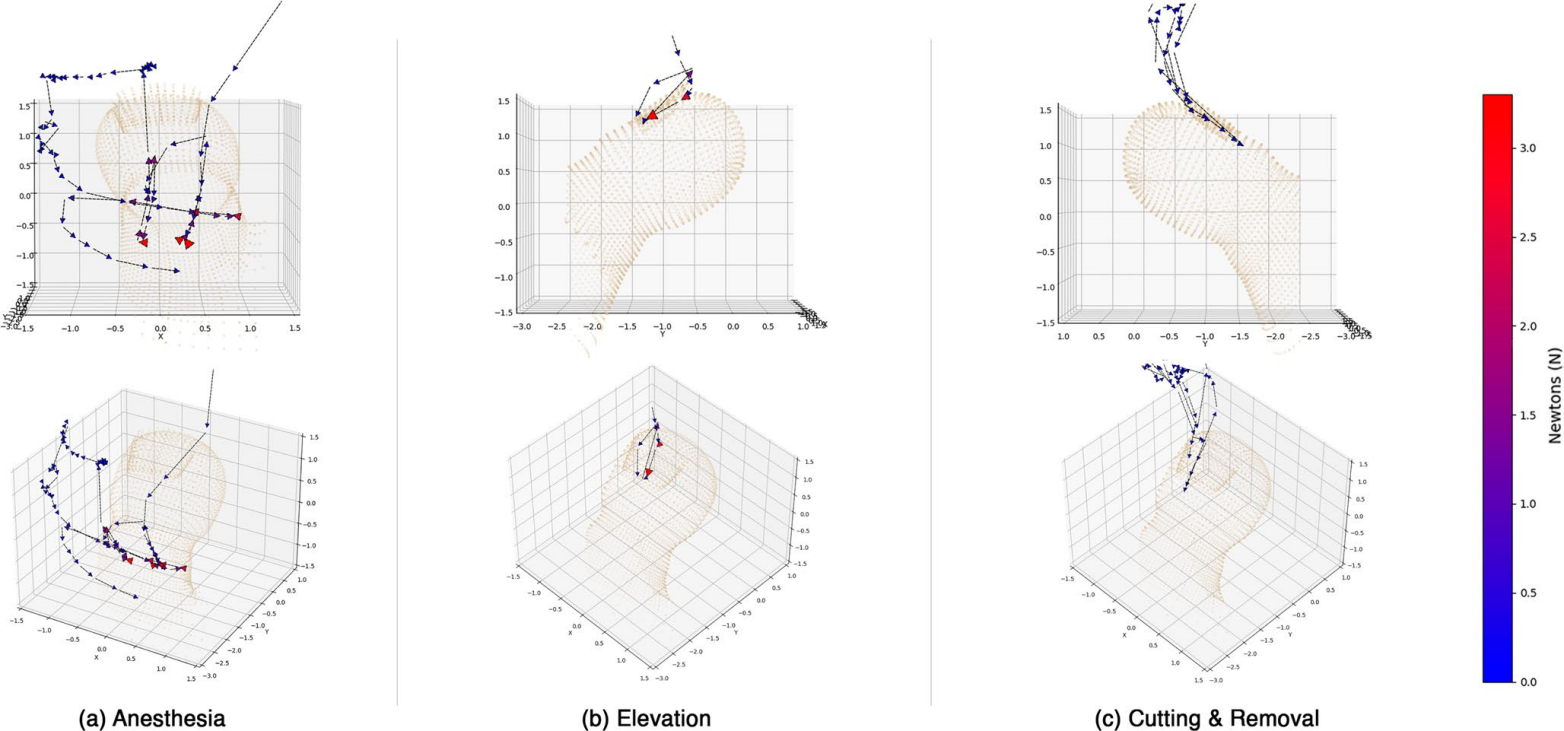
(**a**) 3D Trajectory path of a successful anesthesia attempt on the virtual toe from two perspectives. The big toenail region is faintly illustrated for spatial context. (**b**) 3D Trajectory path of a successful elevation attempt on the virtual toe from two perspectives. (**c**) 3D Trajectory path of a successful cutting and removal attempt on the virtual toe. The density of the dataset was reduced to improve graph clarity

The average completion time per trial was derived and graphed to demonstrate a development of simulation efficiency over each consecutive attempt. It is important that a usable system showcases predictable conditions that can be improved upon. Figure 14a depicts a decreasing trend for both non-medical and medical students in time to complete virtual anesthesia. Non-medical students improved their average completion time by 65.0% from the first attempt. Experienced medical students improved their time by 69.3%. In a similar manner, an increase in elevation task efficiency can be observed by both decreasing trendlines illustrated in Fig. 14b. By the end of the experiment, non-medical students were able to improve their average completion time by 79.5%. Experienced medical students improved their time by 86.3%. Students also improved their completion time for cutting and removal of the ingrown toenail as shown in Fig. 14c. Non-medical students reduced their time to complete by an average of 60.9%. Experienced medical students improved the mean time by 62.2%. We visualize examples of successful task completions in Fig. 15 by plotting the virtual hand-motion data of sample subject over time, of which, showcases the analytical potential of using a virtual surgery simulation.

### System performance

To prove to be a feasible VR-haptic system for educational training, a visually smooth experience is crucial. We investigate the performance impact of our core simulating algorithms by recording the FPS over the duration of the virtual surgery tasks. Table 4 displays the average frame-by-frame performance from a sampled user trial from each task.

**Table 4:** Sample system performance per surgical subtask

Surgical Task	Average FPS	Average Latency	
Anesthesia	67FPS	14.8ms	
Elevation	43FPS	23.4ms	
Cutting & Removal	42FPS	23.9ms	

## Discussion

The initial goal of this study is to evaluate the usability of the VR-haptic system to identify necessary improvements to gear towards creating a potential supplemental learning material for podiatric students. The subjective system usability feedback and objective task performance of medical students give insight into potential student training applicability as well as areas of system usability to improve.

The principal result of the NASA Task Load Index post-assessment result indicates a high perceived level of interactive performance between the user and the system, reflecting a strong alignment between user actions and system response, which meets or satisfies the expectations of the user. As shown in Fig. 9, users did not perceive any of the surgical tasks mentally, physically, or temporally demanding. Users rated low to intermediate mental, physical, and temporal demands as well as perceived effort, and frustration across all surgical tasks. The average perceived workload lies close to the median on the NASA TLX rating scale, suggesting an appropriate difficulty to reward ratio for task completion (i.e. moderate difficulty). Users rated individual subtasks marginally similar on average, further establishing a consistency in the system development. The resulting System Usability Scale highlights key components of a usable surgery simulation system. As shown in Fig. 10, the graph’s resemblance to a 5-pointed star shows strong user agreement with positive system traits such as followed by a disagreement of negative system traits. Agreed upon positive system characteristics suggest high potential usability of the system. User scoring results exhibit strong agreement towards the opinion that the system is well integrated followed by the statement that they would use the system frequently for practice. More so, users disagree that the system is complex, inconsistent, and cumbersome. We consider that some users indicated that they needed to learn a lot or needed support to use the system, where we suggest additional implementation of user interface and experience features to guide users more thoroughly, with less assistance from the study practitioner. Most participants were not medical in this iteration of the study, which may have required more instruction on podiatry procedure, with focus on system usability. In this study, it was noted in Table 3 that some users may not be familiar with VR and significantly fewer were accustomed to haptic technology before being introduced to the simulation for the first time, further suggesting that additional help features may be beneficial to ease the initial learning curve.

The objective user performance data notions a perceptible gap in performance between experienced medical and non-medical students. By comparing the time and pathlength to complete the virtual surgical subtasks of ingrown toenail removal, it can be observed that medical students were able to complete most tasks in less time and shorter hand motion than non-medical students, as portrayed in Figs. 11 and 12. We reject the null hypothesis for completion time between MED and NM groups for anesthesia subtask. In addition, we reject the null hypothesis of virtual anesthesia pathlength between MED and NM groups. The difference in performance may be suggested by past surgical experience. Medical students have learned the operation in podiatric medical school whereas our non-medical student group has not. This demonstrates a potential correlation between skill and performance within the simulation, a necessary correlation for a usable system. On the contrary, medical students took more time and longer hand motion to complete cutting and removal. One possible interpretation for this could be an enhanced sense of carefulness exhibited by medical students due to having real hands-on experience. Nonetheless, when observing user performance by each consecutive attempt, it is shown that the time to complete each task consistently decreases from students’ initial attempt, as shown in Fig. 14. The decline in time and path length over the extent of repeated trials suggests a predictable and consistent learning environment while practicing, which is another necessary component of a usable system. It can be noticed that the variance of the user performance data declines from the initial attempt as participants build experience with each consecutive attempt. The gap in completion time between MED and NM groups seems to shrink and narrow over trials suggesting an interesting relationship caused by repetitive practice. We find that all groups experienced positive improvement in their virtual anesthesia, elevation, cutting, and removal skills from partaking in the experiment. In final analysis, the anesthesia task shows the greatest variation in individual performance, as evidenced by completion time and virtual tool movement distance, as shown in Figs. 11 and 12. These variations result from the relatively challenging perception when inserting a virtual needle into the toe — particularly when haptic cues dominate visual ones, unlike the other two tasks. To explain further, once advancing beyond the skin surface, the tip of the needle is no longer visible. One must primarily rely on physical feeling to ensure the anesthetic needle is fully inserted and removed correctly. This may introduce an additional challenge and provide a learning opportunity for students. Nonetheless, Fig. 14 demonstrates that repeated attempts significantly reduce variations in individual performance due to training with our system. This highlights the system’s usability and potential effectiveness as a training tool, ultimately reducing individual performance discrepancies.

Overall, the evaluation of our preliminary system demonstrates a promising direction in creating a usable tool for improving ingrown toenail removal skills while bringing insight to areas to improve before initiating a longer-term study. We noticed that participants took various time to get used to the system, never using a haptic device before in VR. We suggest additional implementation of built-in guidance features for new users to improve user experience with unfamiliar technology. More success and failure criteria should be defined for each task which may lead to a larger gap between experienced and unexperienced users. There is currently no method to objectively measure the quality of toenail excision. A verification method could be derived from analyzing the 3D hand trajectory with a deep learning model to classify the attempt as a success or failure in a future work. Because the maximum haptic force capable of the devices is 3.3 N, it is possible for users to overpower the device and thus bypass the physical immersion within the simulation. We do not interpret this as an issue with the system as none of the simulated surgical tasks require extensive force, and the limited force ensures a safe environment for the operator in case of an adverse moment of force.

Compared with traditional silicone-based or cadaveric models that naturally preserve realistic interactions between handheld instruments and biological tissues, VR-based haptic systems introduce an additional layer of force-feedback hardware. A potential limitation arises when substituting handheld surgical instruments with stylus-based devices, as this may affect the learning curve and the transfer of hand skills to real environments. To improve usability and more closely approximate clinical practice, future designs could incorporate an engineered interface capable of accommodating multiple surgical instruments (e.g., syringes, hinged instruments, and handles). Despite these limitations, students reported enjoying the experiment process and expressed enthusiasm for the technology. Several participants mentioned they would like to see greater visual realism of the foot with a higher quality 3D model with blood effects. A variety of virtual foot models could create unique training scenarios, each session potentially raising the training difficulty. In respect to the adoption of this new system for future training use, an existing hurdle stands with the institutional deployment of VR surgery simulations as supplemental learning material versus or adjunct to traditional training methods [13]. After making the key improvements, we anticipate launching a comparative study between the VR-haptic system and traditional training methods (i.e., model replica, cadaver) to prove the clear benefit of virtual training in this medical area. And more so, a learning impact study will be performed over multiple virtual training sessions to measure accurate retention. We noticed that some participants’ scores deviated from each other, of which we believe simply taking more time to learn the system, with the future usability improvements, will lead to more consistent scores. The chosen simulated procedure, ingrown toenail removal, aligns well with entry podiatric curriculum to ease the potential transition once proven fully effective for clinical training. We intend to extend the simulation to include other foot and ankle procedures such as bone surgery and joint arthroscopy.

## Conclusion

This work introduces a pivotal step towards a new immersive form of podiatry training by describing a fully functional, ingrown toenail removal simulation in VR with bimanual haptic feedback. A study was conducted to evaluate the usability of the system with both medical and non-medical students. The feedback from users observed through the NASA TLX and SUS suggest positive traits for a usable system. We observed an improvement in completion time, pathlength, and task success of the participants. These results suggest potential applicability for the learning environment. Moving onwards from this work, we will make improvements to enhance graphic and haptic realism, populate the user interface with additional guidance, define extended task success criteria, and then investigate the skill transfer from multiple VR simulation training sessions to a real surgery procedure to validate the material as effect+tive for clinical training.

## Supplementary Information

Below is the link to the electronic supplementary material.

Supplementary File 1 (mp4 174 MB)
